# Research on high school students’ behavior in art course within a virtual learning environment based on SVVR

**DOI:** 10.3389/fpsyg.2023.1218959

**Published:** 2023-07-14

**Authors:** Hongya Wang, Dongning Li, Chao Gu, Wei Wei, Jiangjie Chen

**Affiliations:** ^1^School of Design, Jiangnan University, Wuxi, China; ^2^Department of Culture and Arts Management, Honam University, Gwangju, Republic of Korea; ^3^School of Textile Garment and Design, Changshu Institute of Technology, Changshu, China

**Keywords:** spherical video-based virtual reality, art course, virtual environment, flow, continuance intention

## Abstract

**Introduction:**

Students who use spherical video-based virtual reality (SVVR) teaching materials for learning are able to gain more self-regulated, explorative, and immersive experiences in a virtual environment. Using SVVR teaching materials in art courses can present diverse and unique teaching effects, while also leading to the emergence of students’ flow states.

**Methods:**

Therefore, through an art course teaching experiment, this study investigated 380 high school students and used structural equation modeling to analyze the antecedents and outcomes of students’ flow state in using SVVR teaching materials.

**Results:**

The results show that in using SVVR teaching materials in art courses, more attention should be paid to the control and telepresence in the antecedents of students’ flow state.

**Discussion:**

Only when they obtain better flow experiences can they have higher perceived usefulness and satisfaction with the content of the art course, as well as stronger intentions to continue using it. These results can provide a reference for the development and use of SVVR teaching materials in high school art courses.

## 1. Introduction

In the 2022 edition of the Compulsory Education Art Curriculum Standards issued by the Chinese Ministry of Education, art have been included as a subject for exams in junior and senior high school, and has become a scoring subject for the enrollment of senior high schools. The curriculum also emphasizes the importance of art courses as compulsory subjects (rather than electives) in the nine-year compulsory education. Currently, art education has undergone changes in both its teaching content and methodology due to the development of the times and technological innovations (Tavin and Tervo, 2018; Fan and Zhong, 2022). Fang (2018) suggests that diversifying art curriculum activities can enrich the teaching content of art and improve the teaching effectiveness of art courses. Meanwhile, virtual technology has gradually influenced students’ learning patterns in art education, thus changing the traditional teaching methodology of art courses (Elfeky et al., 2018; Yip et al., 2019; González-Zamar and Abad-Segura, 2020; Wu et al., 2021b). It is evident that virtual technology and digital media serve as powerful and important supplementary teaching tools in art education, with enormous potential (Leonard, 2020; Gong, 2021; Gu et al., 2022; Jiang et al., 2022).

Previously, art education research has seen the emergence of many new directions, such as visual experience (Marosi, 2021), scenario-based learning environments (Gillespie, 2018), and self-regulated learning (Wu et al., 2021b), among others. This implies that the teaching methodology in art education has undergone a corresponding change, shifting from a teacher-centered, skills-based mode to a student-centered one that encourages skill development through exploration and interaction (Fan and Zhong, 2022). Moreover, the widespread use of virtual technology in education has transformed teachers from mere knowledge transmitters to facilitators and guides of student learning, while students have transitioned from passive receivers of knowledge to creators and innovators (Sanabria and Arámburo-Lizárraga, 2017; Du et al., 2022). Hence, the emerging virtual technology and innovative learning methods have not only addressed the limitations of traditional teaching methods but also provided students with immersive virtual learning environments and rich interactive learning content, empowering them to engage in self-directed exploratory learning and experiences (Johnson et al., 2020; Chen et al., 2021).

Virtual Reality (VR) have three major characteristics: immersion, interaction, and imagination (Burdea and Coiffet, 2003). These three characteristics of VR technology enable users to experience a high degree of immersion, realism, and interactivity in their perception of the simulated environment, which allows them to trust the environment they are in (Wu et al., 2020). The virtual learning environment with visualizations can encourage students to explore intuitively and engage in interactive learning with the virtual environment driven by their curiosity (El et al., 2019; Natale et al., 2020). Hence, virtual technology-based teaching aids stand out in various educational fields (Makransky et al., 2019; Radianti et al., 2020; Luo et al., 2021). However, the application of VR teaching materials in education has become a challenge for many universities or learners due to the high cost of investment, and the development of VR teaching materials is not easy (Esteban-Millat et al., 2014; Geng et al., 2021). Spherical Video-based Virtual Reality (SVVR) is a tool that provides spherical images and videos for users to obtain knowledge content in a virtual learning environment. SVVR has not only become an easy-to-implement and cost-effective learning tool in teaching classrooms, but also a potential technology for students to engage in experiential learning activities (Chiu et al., 2022). In addition, due to its simple development and operation of teaching materials, SVVR is not only easy to promote in basic education (Yang et al., 2022), but also enables students to break through the constraints of time and space, and more effectively utilize learning resources whether in-class or after-class (Yang et al., 2022).

It is worth noting that when students learn through virtual environments generated by AR/VR technology, it often accompanies the generation of flow (Ibáñez et al., 2014). The concept of flow can be understood as a psychological state of complete engagement, enjoyment, and the loss of sense of time and space (Shin, 2018, 2019). This immersive experience not only enhances students’ engagement, but also encourages students to enter the optimal flow state (Chen and Hsu, 2020; Gu et al., 2022; Tai et al., 2022). Therefore, using AR/VR virtual environments in teaching modes has significant advantages over traditional modes, and students’ flow state is an important factor that cannot be ignored in the use of SVVR teaching materials. Regarding the previous literature findings, there is limited research on the use of SVVR teaching materials for learning in art courses, and there are also significant shortcomings. Therefore, this study aims to explore the flow experience of high school students when using SVVR teaching materials for learning in art courses, with the purpose of investigating the factors that influence students’ continuous use of SVVR teaching materials in art course learning. The results of this study can provide reference for the development of SVVR teaching materials in future art courses.

## 2. Literature review

### 2.1. SVVR in art curricula

The experimentation with VR technologies in primary, secondary, and higher education began in the early 1990s (Youngblut, 1998). However, in the early days of using VR for learning, the equipment was rather complex, requiring students to don head-mounted displays, data gloves, and body suits. Nowadays, there have been several important categories of Virtual reality, including desktop VR, immersive VR, distributed VR and augmented VR (Sun et al., 2018). SVVR belongs to the category of immersive VR, and is comparatively more portable and user-friendly than head-mounted VR devices. Head-mounted VR devices can simulate real-life environments using computer technology, providing users with an interactive experience with virtual objects (Shen et al., 2019). The inherent features of VR allow students to interact with learning materials, thereby improving their learning outcomes (Kang et al., 2022). Additionally, 360-degree video and VR technologies can provide a comprehensive immersive experience for classroom teaching, which not only gives teachers greater control over classroom interactions but also increases their willingness to use SVVR in teaching (Cross et al., 2022).

Obviously, SVVR has vast potential for applications in the field of education. On the one hand, SVVR can provide students with an immersive learning experience that traditional textbooks cannot achieve in terms of visual and sensory stimuli (Moro et al., 2017; Degli et al., 2019). On the other hand, SVVR allows students to be present in virtual learning environments to engage with learning materials and easily navigate to view knowledge they wish to learn (Walshe and Driver, 2019). In the past 5 years, SVVR has become a popular research topic in various educational fields (Sun et al., 2018; Wu et al., 2019, 2021b; Huang et al., 2020; Chiu et al., 2022). Scholars have conducted research on the application of SVVR in various art education settings, including music (Degli et al., 2019), art museums (Chiu et al., 2022), landscape architecture design (Wu et al., 2021a), and more.

Typically, high school art classes focus on cultivating students’ aesthetic and independent exploration awareness, as well as promoting critical and creative thinking and artistic practice skills through art appreciation (Walker, 2014; Artz, 2021). Therefore, the application of SVVR in virtual learning environments in art classrooms is in line with the current emphasis on visual experience and encouraging exploration in art education. Furthermore, as self-directed learning gains increasing attention in the research on factors affecting learning achievement, deeper investigations are being carried out on its relationship with VR education (Liu et al., 2022). Therefore, this study deems it meaningful to delve into the implementation of SVVR teaching materials in art curriculum during the secondary education level.

### 2.2. Flow experience

According to flow theory, flow is considered as the optimal experience (Csıkszentmihályi, 1975), where individuals become immersed in the activity at hand and forget about themselves, leading to a pleasurable psychological state (Csıkszentmihályi, 1975; Csikszentmihalyi, 1990). This psychological state enables individuals to be fully engaged in an activity without self-awareness, allowing learners to participate in a learning environment according to their intrinsic motivation (Csikszentmihalyi, 1990). Learners may temporarily lose their self-consciousness and sense of time, as all their mental energy is focused on their online learning and interaction (Esteban-Millat et al., 2014). Hence, there is clear agreement among the literature on the concept of flow (Engeser et al., 2021). The flow state is intrinsically rewarding, meaning that individuals seek to replicate flow experiences (Csikszentmihalyi et al., 2014). The flow experience can motivate students to develop sufficient attention towards virtual learning environments and derive pleasure through learning activities (Faiola et al., 2013). When students experience flow, students are motivated not by the potential rewards of completing a specific task but by the gratifying sensation derived from performing the task (Esteban-Millat et al., 2018).

For instance, Gao et al. (2019) refers to a framework for designing learning activities that can be implemented in a smart learning environment. Through the experience of flow, students can enhance their immersion and participation, and gain a pleasurable learning experience. In the research conducted by Chen and Hsu (2020), it was found that the interactive features of VR applications and the challenges presented by game-based design enable students to easily enter a state of flow and increase their motivation to learn. Therefore, scholars suggests that the flow is a factor that affects students’ learning state in virtual environments (Sun et al., 2017; Rodríguez-Ardura and Meseguer-Artola Rodriguez-Ardura and Meseguer-Artola, 2019). Furthermore, students’ state of flow is also considered a key factor in promoting high-quality learning attitudes and participation in virtual learning environments (Bodzin et al., 2021).Recent research has found that in addition to the education field, flow theory has been proven applicable in various fields such as gaming (Yen and Lin, 2022), leisure and entertainment (Leung, 2020), online social shopping (Tuncer, 2021), sports activities (Kim and Ko, 2019), and work (Kawalya et al., 2019). Furthermore, research on flow theory based on VR technology has found that VR can create immersive virtual environments for users and induce their experience of flow and engagement (Pallavicini and Pepe, 2019; Huang et al., 2021). In VR virtual learning environments, research has found that students’ self-directed learning has a significant impact on learning attitudes and can improve satisfaction and the intention to continuous use by enhancing students’ experience of flow.

Previous research has indicated that the emergence of the state of flow can be divided into three stages: flow antecedents, flow experience, and flow consequences (Finneran and Zhang, 2003; Liu and Song, 2021). Initially, flow antecedents refer to the prerequisites that trigger the state of flow in users, such as the user’s perceived challenge, skill level, and clarity of goals related to the task at hand (Hamari et al., 2016; Wan et al., 2021). Then, flow experience is the user’s virtual immersive experience during the task process (Zhao and Khan, 2022). Lastly, flow consequences are the positive outcomes experienced by users after the occurrence of the state of flow, such as positive attitudes (Bodzin et al., 2021), satisfaction with learning (Zhao and Khan, 2022), and increased intention to continue learning (Rodriguez-Ardura and Meseguer-Artola, 2019). Therefore, the proposed model of this study will be divided into three stages: flow antecedents, flow experience, and flow consequences, to analyze and explore the factors that influence students’ sustained use of SVVR for learning in an art course.

### 2.3. Research hypotheses

#### 2.3.1. Flow antecedents in SVVR

In the education-related literature, the factors that influence the flow antecedents of students in different learning environments vary (Huang et al., 2010; Esteban-Millat et al., 2014; Guo et al., 2016; Rodríguez-Ardura and Meseguer-Artola, 2016; Rodríguez-Ardura and Meseguer-Artola, 2017; Mulik et al., 2019; Liu and Song, 2021). For example, knowledge workers believe that when they feel a complete lack of control during knowledge sharing, they are unlikely to be focused on the task at hand, resulting in a weakened flow experience (Lin and Joe, 2012). In this study, operational definition of control is described as the sense of control that students have when engaging in interactive learning with SVVR teaching materials in a virtual environment (Koufaris, 2002). In other words, a high level of control over the knowledge content can enhance the learning experience (Park et al., 2010; Barthelmäs and Keller, 2021) and influence the flow experience (Rodríguez-Ardura and Meseguer-Artola, 2016). Studies have shown that student participation and learning outcomes can be improved through self-directed control in VR, leading to the experience of flow while playing VR learning games (Bodzin et al., 2020). Therefore, this study considers the perceived control of students during SVVR-based learning as a prerequisite factor influencing the flow experience.

Additionally, a central component of flow is the perceived balance between the demands of the task and the individual’s skills (Csıkszentmihályi, 1975). The level of self-efficacy is an individuals’ evaluation of his/her skills and therefore has a substantial impact on how the balance between skills and task demands is perceived (Peifer et al., 2020). The operational definition of self-efficacy in this study is the level of confidence that students have when engaging in interactive learning with SVVR teaching materials (Carroll, 2002). Rodríguez-Sánchez et al. (2011) found that the channel model of flow, including self-efficacy as antecedent of flow, could better fit the data，and discovered that teachers’ work-related self-efficacy positively affected their flow experience. In online learning environments, the more learners believe in their ability to successfully complete learning tasks, the more actively they will participate in the learning process and experience a state of flow (Joo et al., 2015). Flow is characterized by self-efficacy, or the perception of challenge that arises during interaction, resulting in a positive experience of total engagement when the challenge is of moderate difficulty (Hamari et al., 2016). In the academic setting, Mesurado et al. (2016) verified that self-efficacy promotes both flow experience and study engagement in students. It can be considered that the strength of self-efficacy has a positive impact on the flow experience.

Furthermore, this research defines the operational concept of “telepresence” as the immersive sensation experienced by students when utilizing SVVR instructional materials (Steuer et al., 1995). Generally, telepresence describes the user’s experience of the virtual reality environment (Held, 1992; Steuer et al., 1995; Kim and Biocca, 1997), as the level of telepresence becomes higher, the experience of virtual reality environment becomes more elaborated(Klein, 2003). Telepresence is also deemed a determining factor in generating flow experiences (Skadberg and Kimmel, 2004; Shin, 2006; Zaman et al., 2010). For example, Telepresence helps users to forget the real world and concentrate on the virtual world (Zaman et al., 2010), and further strengthens users’ flow experience (Li and Peng, 2021). In the virtual learning environment, the study highlights the crucial role of students’ sense of “presence” in eliciting flow experiences, thereby enhancing their learning experiences (Huang et al., 2010; Guo et al., 2016). Furthermore, studies have mentioned that telepresence will significantly influence the flow experience of users in different virtual scenarios (Kim and Ko, 2019; Liu et al., 2023). Therefore, this study hypothesizes that students are more likely to enter a state of flow in virtual learning environments through a sense of telepresence.

In summary, this study posits that the prerequisite factors for generating flow experiences among students engaged in interactive learning using SVVR teaching materials in art classes are control, self-efficacy, and “presence,” and puts forth the following hypotheses:

*Hypothesis 1 (H1)*: The level of control that students possess while utilizing SVVR teaching materials for learning has a significant positive impact on the experience of flow.

*Hypothesis 2 (H2)*: The degree of self-efficacy that students feel while using SVVR teaching materials for learning has a significant positive impact on the experience of flow.

*Hypothesis 3 (H3)*: The level of telepresence that students feel while using SVVR teaching materials for learning has a positive significant impact on the experience of flow.

#### 2.3.2. Flow experience in SVVR

The present study operationalizes the concept “perceived usefulness” as the extent to which students perceive that their use of SVVR instructional materials enhances their learning efficiency (Davis, 1989). Liaw (2008) indicates that perceived usefulness, in combination with multimedia quality, is a significant predictor of e-learning effectiveness. Additionally, Obeid and Demirkan (2020) show that participants who use virtual design environments during the design process experience a sense of participation and heightened flow states, and are able to attain greater motivation to learn. Therefore, in the realm of virtual technology applications in education, scholars have noted a higher perceived usefulness of AR/VR technology when employed by teachers during instruction (Alalwan et al., 2020; Jang et al., 2021).

Generally, the operational definition of satisfaction is the sense of accomplishment that users obtain from their actual experiences compared to their expected experiences (Hernon and Whitman, 2001). In this study, satisfaction is regarded as the emotional state produced by students when using SVVR instructional materials. For instance, in online learning environments, the state of flow experienced by students has an impact on their satisfaction with the use of MOOCs platform (Shanshan and Wenfei, 2022). Additionally, in the context of virtual reality technology, media users’ satisfaction is determined by a series of discrete experiences such as cognitive absorption, time distortion, and enjoyment (Kim and Ko, 2019). Furthermore, numerous studies have suggested that users’ flow experience has a positive effect on their satisfaction in research related to user behavior and new media technologies (Chang and Zhu, 2012; Lee and Choi, 2013; Kim and Ko, 2019; Mulik et al., 2019).

The present study defines the operational definition of continuance intention as students’ willingness to engage in interactive learning using SVVR teaching materials (Bhattacherjee, 2001). In the context of online learning environments, students who experience a better state of flow are more likely to have a higher intention to continue using virtual classrooms (Li et al., 2022). Previous research has suggested that the positive impact of flow on continuous usage intention is evident when students use language learning applications (Wang et al., 2022). Therefore, this study posits that when students use SVVR teaching materials for learning and experience a state of flow, they perceive SVVR material as an effective tool for learning, which improves their satisfaction and willingness to continue using SVVR teaching materials for learning tasks. Based on this, the study puts forth the following hypotheses:

*Hypothesis 4 (H4)*: The state of flow experienced by students when using SVVR teaching materials for learning has a significant positive impact on perceived usefulness.

*Hypothesis 5 (H5)*: The state of flow experienced by students when using SVVR teaching materials for learning has a significant positive impact on satisfaction.

*Hypothesis 6 (H6)*: The state of flow experienced by students when using SVVR teaching materials for learning has a significant positive impact on continuance intention.

#### 2.3.3. Flow consequences in SVVR

In the Post-Acceptance Model of IS proposed by Bhattacherjee (2001), it was found that the perceived usefulness of adopting an information system has a positive impact on users’ satisfaction and intention to continue using the system. Furthermore, users’ satisfaction with the system has a positive impact on their intention to continue using it. In the field of education, Liaw and Huang (2013) found that perceived satisfaction toward e-learning is a contributor to the perceived usefulness of e-learning. Zhang et al. (2017) and Wang et al. (2022) have reported that perceived usefulness of language learning applications or virtual learning communities has a positive impact on continuous usage intention. In online learning environments, student satisfaction has been found to significantly affect their intention to continue using the system (Koufaris, 2002; Shanshan and Wenfei, 2022).

The research on using VR technology for learning tasks has shown that the perceived usefulness of the user significantly affects their intention to use it (Sagnier et al., 2020). Some studies have also suggested that perceived usefulness has a positive impact on the intention to continue using it (Hou et al., 2018). As Du et al. (2022) have demonstrated, teachers using VR technology to simulate immersive interactive learning environments can effectively support teaching tasks and increase their intention to continue using VR technology in the future for teaching. Furthermore, in learning environments supported by new media technology, learners’ satisfaction can increase their intention to continue using the learning platform. In the field of AR technology, research by Kim et al. (2016) has indicated that the perceived usefulness of using MAR apps significantly affects satisfaction and the intention to continue using it, and users’ satisfaction strongly influences their intention to continue using it. At the same time, research has shown that students’ satisfaction with using AR guides in museums has a positive effect on their intention to continue using it (Jiang et al., 2022). Therefore, this study proposes the following hypotheses:

*Hypothesis 7 (H7)*: The perceived usefulness of using SVVR instructional materials for student learning has a significant positive impact on their satisfaction.

*Hypothesis 8 (H8)*: The perceived usefulness of using SVVR instructional materials for student learning has a significant positive impact on their continuance intention.

*Hypothesis 9 (H9)*: Students’ satisfaction with using SVVR instructional materials for learning has a significant positive impact on their continuance intention.

**Figure 1:**
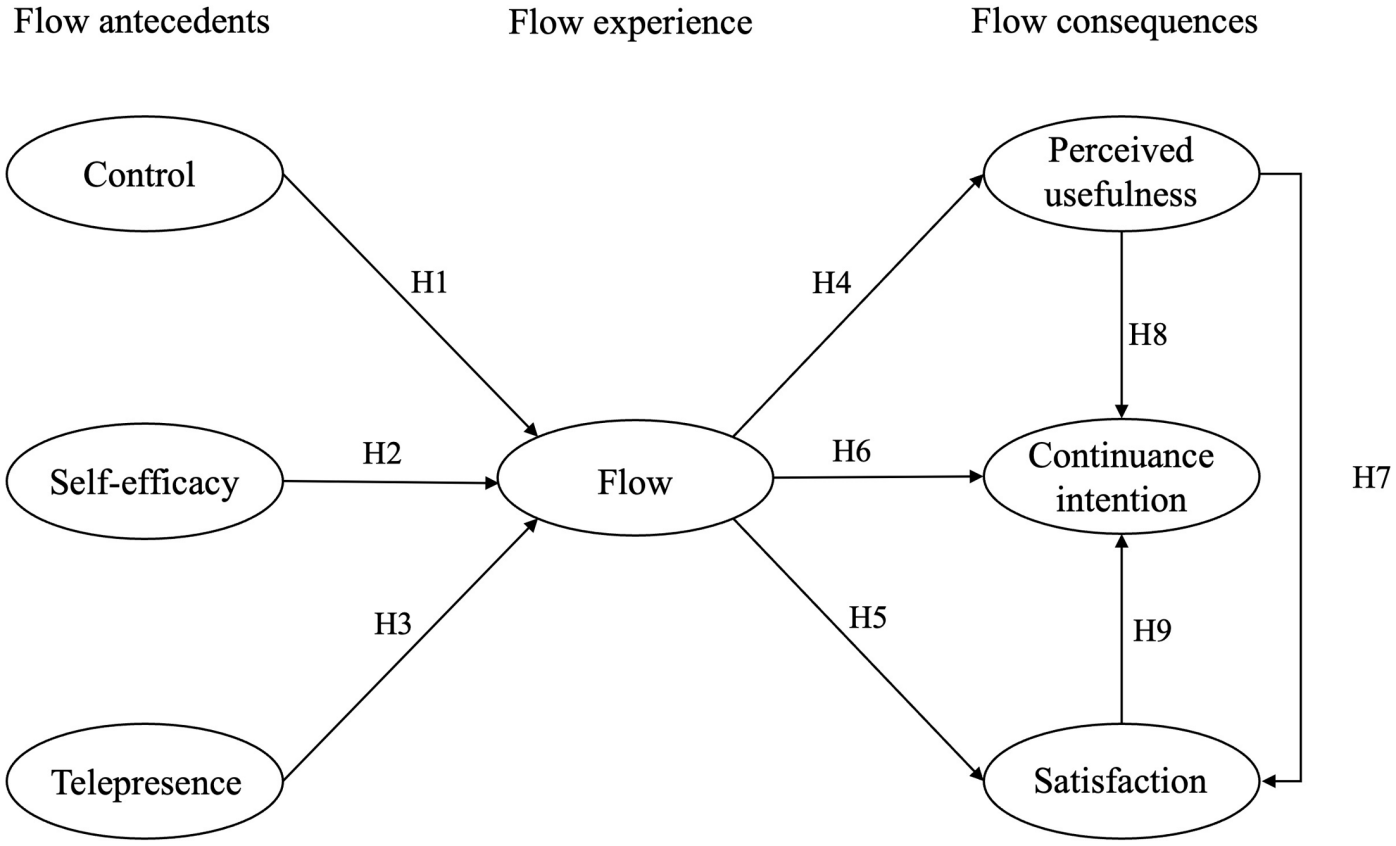
Research model.

This study first assumes from the theory of flow experience that the antecedent variables control, self-efficacy, and telepresence have a direct impact on the production of flow when students use SVVR teaching materials for learning in art courses, as well as an indirect impact on the outcome variables of flow experience, including perceived usefulness, satisfaction, and continuance intention. Secondly, this study assumes that perceived usefulness has an impact on continuance intention through its influence on satisfaction, and that satisfaction has a direct impact on continuance intention. Therefore, this study proposes the following research hypothesis model, as shown in Figure 1.

## 3. Methodology

### 3.1. Research design

A total of 380 Chinese high school students, including 215 male students (56.6%) and 165 female students (43.4%), participated in the teaching experiment, all of whom had prior experience using VR/AR devices. The teaching experiment lasted 2 weeks (students took class once a week), the students used SVVR teaching materials in a virtual environment based on the teacher’s course content to learn about specific art-related topics. The SVVR learning materials used in the art course were obtained from the UtoVR website.^1^ After the course, students were invited to complete a survey questionnaire. Moreover, this study was conducted in accordance with the guidelines of the Declaration of Helsinki and received ethical approval from the review committee of the Ministry of Social Science, Changshu Institute of Technology. Informed consent was obtained from all participants, and all methods were carried out in accordance with relevant guidelines and regulations.

### 3.2. Questionnaire design

The questionnaire items used for this study was adapted from scales validated in previous research, and was set as Likert’s 7-point style. During the pre-test phase of the questionnaire survey, five respondents who fit the scope of this study were randomly invited to answer and provide modifying feedback of the questionnaire to improve its readability. Reverse questions were designed in the questionnaire to check the validity of the respondents’ answers, and respondents’ focus was assessed through their response times. The operational definitions of the variables and items are shown in Table 1.

**Table 1:** Questionnaire items.

Operational definition of variables and content of questions	Source
Control The sense of control that students have when engaging in interactive learning with SVVR teaching materials in a virtual environment When using SVVR instructional materials, … CO1 I would feel a sense of control CO2 I believe there are advantages. CO3 I feel a sense of self-regulated. CO4 I have a clear understanding of what needs to be done.	Koufaris (2002)
	Esteban-Millat et al. (2014)
Self-efficacy The level of confidence that students have when engaging in interactive learning with SVVR teaching materials. When using SVVR instructional materials, … SE1 I can resolve any problem in learning. SE2 I have confidence in myself to master new knowledge. SE3 I believe I can overcome any difficulties I encounter. SE4 I am confident that I can accomplish any challenging academic tasks I encounter. SE5 I always manage to find strategies to overcome new academic challenges.	Carroll (2002)
	Hong et al. (2017)
Telepresence The immersive sensation experienced by students when utilizing SVVR instructional materials When using SVVR instructional materials, … TE1 I feel as if I am immersed in a virtual environment. TE2 I find that virtual environments can be more realistic than real-life environments. TE3 Although I am physically present in the classroom, my mind feels as if it is within a virtual environment. TE4 I have noticed that the virtual environment created by the SVVR teaching materials has disappeared. TE5 I feel as if I have returned to the real-life environment.	Steuer et al. (1995)
	Hou et al. (2018), Pelet et al. (2017)
Flow When students utilize SVVR teaching materials with complete concentration, they often forget the passage of time and immerse themselves in the process, resulting in a state of flow experience. When using SVVR instructional materials，···· FL1 I feel as if time has passed quickly FL2 I feel very curious. FL3 I have not thought about anything else. FL4 I am completely captivated.	Csıkszentmihályi (1975)
	Cheng (2020), Esteban-Millat et al. (2014)
Perceived usefulness The extent to which students perceive that their use of SVVR instructional materials enhances their learning efficiency. PU1 The SVVR teaching materials has been very beneficial for my learning. PU2 The SVVR teaching materials make it convenient for me to access the study content. PU3 The SVVR teaching materials can improve my learning efficiency.	Davis (1989)
	Al-Emran et al. (2020), Makransky and Lilleholt (2018)
Satisfaction the sense of accomplishment that users obtain from their actual experiences compared to their expected experiences. SA1 I am satisfied with my learning experience using the SVVR teaching materials. SA2 I am satisfied with the teaching method utilized in the SVVR teaching materials. SA3 I do not believe that using the SVVR teaching materials has helped me to understand the study content. ® SA4 I believe that the SVVR teaching materials have met my expectations. SA5 I am satisfied with the overall learning outcome of using the SVVR teaching materials.	Doll et al. (1998)
	Al-Emran et al. (2020), Makransky and Lilleholt (2018)
Continuance intention Students’ willingness to engage in interactive learning using SVVR teaching materials CI1 I plan to continue using the SVVR teaching materials for my future studies. CI2 I plan to regularly use the SVVR teaching materials for my future studies. CI3 Overall, I plan to continue using the SVVR teaching materials for my studies.	Bhattacherjee (2001)
	Ashrafi et al. (2020), Al-Emran et al. (2020)

®Reverse coded; all items used in this study were measured on a 7-point Likert’s scale ranging from strongly disagree (1) to strongly agree (7).

## 4. Results and discussion

### 4.1. Reliability test

Reliability test of this study was conducted by IBM SPSS software, and the results are shown in Table 2. The corrected item total correlations between each construct and its corresponding items were all above 0.5, indicating that the scores of the items within each construct were similar to each other (Zijlmans et al., 2019). In addition, all constructs passed tests of reliability and validity after the removal of items CO1, SE1, TE4, TE5, FL2, SA3, and SA4. The reliability of each construct was greater than 0.7, and no further improvement in reliability was observed after the removal of any item. Overall, the data collected in this study was reliable and suitable for further analysis.

**Table 2:** Results of reliability test.

Construct	Items	Corrected item total correlation	Cronbach’s alpha if item deleted	Cronbach’s alpha
Control	CO2	0.630	0.712	0.788
	CO3	0.634	0.706	
	CO4	0.624	0.720	
Self-efficacy	SE2	0.650	0.823	0.849
	SE3	0.684	0.810	
	SE4	0.736	0.786	
	SE5	0.680	0.811	
Telepresence	TE1	0.658	0.616	0.759
	TE2	0.554	0.745	
	TE3	0.587	0.683	
Flow	FL1	0.570	0.722	0.768
	FL3	0.580	0.712	
	FL4	0.655	0.629	
Perceived usefulness	PU1	0.720	0.761	0.840
	PU2	0.690	0.791	
	PU3	0.704	0.779	
Satisfaction	SA1	0.782	0.748	0.856
	SA2	0.707	0.819	
	SA5	0.699	0.827	
Continuance intention	CI1	0.842	0.838	0.905
	CI2	0.772	0.896	
	CI3	0.819	0.856	

### 4.2. Exploratory factor analysis

The study adopted exploratory factor analysis to test the unidimensionality of the data, and the results are presented in Table 3. Principal component analysis was used as the specific calculation method, and varimax was used for rotation. To verify whether the data was suitable for exploratory factor analysis, this study conducted Kaiser-Meyer-Olkin (KMO) test and Bartlett’s sphere test. The results showed that the KMO values for each construct were greater than 0.5, and the significance of Bartlett’s sphere test was less than 0.05, which indicates that there were partial correlations between items and rejects the null hypothesis that the correlation matrix is an identity matrix. Therefore, the conduction of exploratory factor analysis was deemed appropriate (Abdullah et al., 2020).

**Table 3:** Results of exploratory factor analysis.

Construct	Items	KMO	Bartlett’s sphere test	Commonality	Factor loading	Eigenvalue	Total variation explained
Control	CO2	0.707	0.000*	0.704	0.839	2.110	70.331%
	CO3			0.708	0.842		
	CO4			0.697	0.835		
Self-efficacy	SE2	0.815	0.000*	0.644	0.802	2.752	68.800%
	SE3			0.684	0.827		
	SE4			0.745	0.863		
	SE5			0.679	0.824		
Telepresence	TE1	0.682	0.000*	0.748	0.865	2.059	68.624%
	TE2			0.629	0.793		
	TE3			0.682	0.826		
Flow	FL1	0.686	0.000*	0.650	0.806	2.054	68.458%
	FL3			0.663	0.814		
	FL4			0.741	0.861		
Perceived usefulness	PU1	0.727	0.000*	0.774	0.880	2.275	75.833%
	PU2			0.744	0.863		
	PU3			0.757	0.870		
Satisfaction	SA1	0.717	0.000*	0.829	0.910	2.331	77.706%
	SA2			0.756	0.870		
	SA5			0.746	0.864		
Continuance intention	CI1	0.744	0.000*	0.870	0.933	2.523	84.089%
	CI2			0.803	0.896		
	CI3			0.850	0.922		

* The level of significance is 0.05.

This study attempted to extract new factors with eigenvalues greater than 1 for each of the constructs. The results indicate that only one new factor could be extracted from each construct, and the total variation explained was greater than 60%, which suggests that there are no sub-concepts within the constructs, and the new factors can explain the original items well. Furthermore, all items had a commonality greater than 0.5 and a factor loading greater than 0.6, indicating that the items within the same construct are correlated, which meets the criteria suggested in previous studies (Kohli et al., 1998). In conclusion, the data used for analysis met the requirements of unidimensionality.

### 4.3. Confirmatory factor analysis

The study examined the convergent validity and discriminant validity of the constructs to determine whether the items have acceptable factor loadings on their corresponding constructs and whether there is excessive correlation between the constructs, which may lead to collinearity issues. The results are presented in Table 4. All the fit indices of the confirmatory factor analysis met the recommended standards from previous research, indicating a good fit of the CFA model (Hair et al., 2006). Additionally, we calculated the common latent factor method (CCLFM) to establish a control model to test the common method bias. we found that there was no significant change in indexes between CCLFM and CFA based on the results of model fitting. Among them, the change in RMSEA of CCLFM is less than 0.05, and the changes in GFI, TLI, NFI, CFI, and SRMR do not exceed the critical value of 0.1. This indicates that the model did not show significant improvement after introducing the common method factor. Therefore, the problem of common method bias in this study has been well controlled, and we found no apparent method bias in the data (Rajesh, 2021).

**Table 4:** Measures of fit for CFA and CCLFM.

Common indices	*χ*^2^/df	RMSEA	GFI	TLI	NFI	CFI	SRMR
Judgment criteria	<5	<0.08	>0.9	>0.9	>0.9	>0.9	<0.08
CFA Value	1.759	0.045	0.923	0.967	0.940	0.973	0.030
CCLFM Value	1.758	0.045	0.925	0.967	0.940	0.973	0.035

*χ*^2^/DF = normed Chi-square, RMSEA = root mean square error approximation, GFI = goodness of fit index, TLI = tucker-lewis index, NFI = normative fit index, CFI = comparative fit index, SRMR = standardized root mean square residual.

The results of the confirmatory factor analysis indicate that the factor loadings of all items are greater than 0.6, and the squared multiple correlation (SMC) results are greater than 0.4, which meets the standards suggested in previous studies (Adaileh et al., 2020). Moreover, the average variance extracted (AVE) of each construct is greater than 0.5, indicating that all items in each construct consistently explain the construct (Chung et al., 2020). Additionally, the results of composite reliability (CR) are all greater than 0.7, which are considered acceptable and demonstrate the combination reliability, as shown in Table 5. Hence, this study posits that the constructs in the model have convergent validity.

**Table 5:** Results of convergent validity test.

Construct	Items	Factor loading	SMC	*t* value	S.E.	*p* value	AVE	CR
Control	CO2	0.757	0.572	16.364	0.028	0.001*	0.558	0.791
	CO3	0.733	0.537	15.676	0.028	0.001*		
	CO4	0.745	0.554	16.010	0.030	0.002*		
Self-efficacy	SE2	0.722	0.521	15.564	0.028	0.001*	0.586	0.850
	SE3	0.758	0.575	16.680	0.029	0.001*		
	SE4	0.812	0.659	18.409	0.024	0.001*		
	SE5	0.767	0.589	16.963	0.030	0.002*		
Telepresence	TE1	0.776	0.602	16.804	0.035	0.002*	0.534	0.774
	TE2	0.686	0.471	14.273	0.034	0.001*		
	TE3	0.730	0.533	15.492	0.035	0.001*		
Flow	FL1	0.748	0.559	16.083	0.033	0.001*	0.526	0.768
	FL3	0.654	0.428	13.501	0.037	0.001*		
	FL4	0.771	0.594	16.751	0.031	0.001*		
Perceived usefulness	PU1	0.835	0.697	19.314	0.024	0.001*	0.641	0.843
	PU2	0.758	0.575	16.783	0.026	0.001*		
	PU3	0.799	0.639	18.107	0.025	0.001*		
Satisfaction	SA1	0.848	0.719	19.777	0.029	0.002*	0.668	0.857
	SA2	0.793	0.629	17.895	0.024	0.002*		
	SA5	0.813	0.660	18.552	0.026	0.001*		
Continuance intention	CI1	0.906	0.822	22.426	0.017	0.002*	0.764	0.907
	CI2	0.821	0.674	19.144	0.020	0.001*		
	CI3	0.893	0.797	21.877	0.018	0.001*		

* The level of significance is 0.05.

This study adopted the Fornell-Larcker criterion method to examine the discriminant validity between constructs (Fornell and Larcker, 1981), as shown in Table 6. This method compares the square roots of AVE with the Pearson correlation coefficients between constructs to determine discriminant validity. The results show that the square roots of AVE for each construct are greater than the correlation coefficients between any of the correlation coefficients, indicating that the constructs have good discriminant validity. In addition, as shown in Table 7, the Heterotrait- Monotrait (HTMT) ratio shows good scores, almost all below 0.9, which are acceptable (Gold et al., 2001). There is only one value slightly larger 0.9 that occurs in the relation between FL and TE. In this case, we have performed a bootstrapping with 5,000 subsamples to calculate the HTMT inference value to test discriminant validity, and finally recruited that confidence interval does not contain 1 as judgement criterion (Ramírez-Correa et al., 2019). The results show that the confidence interval varies between 0.844 and 0.977, as value one is outside this range, we can suggest that the FL and TE constructs are empirically distinct (Henseler et al., 2015).

**Table 6:** Discriminant validity: Fornell-Larcker criterion.

	CO	SE	TE	FL	PU	SA	CI
CO	**0.747**						
SE	0.705*	**0.767**					
TE	0.570*	0.618*	**0.731**				
FL	0.598*	0.603*	0.702*	**0.725**			
PU	0.667*	0.635*	0.592*	0.615*	**0.801**		
SA	0.635*	0.617*	0.600*	0.606*	0.748*	**0.817**	
CI	0.632*	0.645*	0.634*	0.665*	0.724*	0.710*	**0.874**

* The level of significance is 0.05. The bolded values on the diagonal are the square root values of AVE values.

**Table 7:** Discriminant validity: Heterotrait-Monotrait ratio (HTMT).

	CO	SE	TE	FL	PU	SA	CI
CO							
SE	0.861						
TE	0.732	0.762					
FL	0.767	0.747	0.911				
PU	0.819	0.752	0.739	0.765			
SA	0.774	0.724	0.746	0.747	0.882		
CI	0.747	0.735	0.760	0.797	0.828	0.806	

### 4.4. Model fit test

The present study used IBM AMOS software to establish the structural equation model and validate the hypothesized model using the maximum likelihood method with 2000 times bootstrap and 95% confidence interval (Hair et al., 2006). The model fit indices are shown in Table 8, which show that all indices meet the recommended standards.

**Table 8:** Results of model fit test.

Common indices.	*χ*^2^/df	RMSEA	GFI	TLI	NFI	CFI	SRMR
Judgment criteria	< 5	< 0.08	> 0.9	> 0.9	> 0.9	> 0.9	< 0.08
Value	1.866	0.048	0.915	0.962	0.933	0.967	0.036

*χ*^2^/DF = normed Chi-square, RMSEA = root mean square error approximation, GFI = goodness of fit index, TLI = tucker-lewis index, NFI = normative fit index, CFI = comparative fit index, SRMR = standardized root mean square residual.

**Figure 2:**
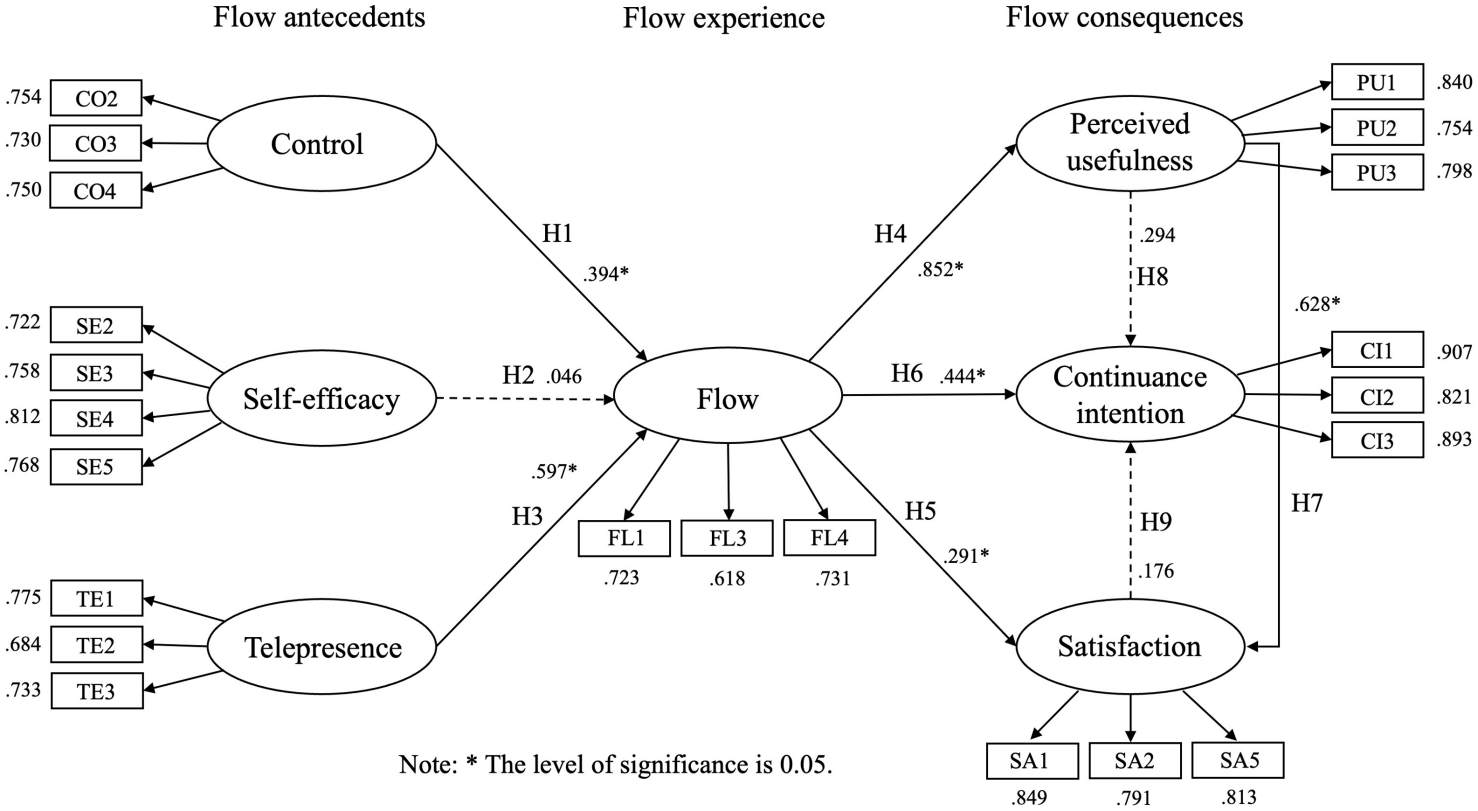
Analysis results of hypothesized model.

The path analysis results of the present study are shown in Table 9. The results indicate that, except for the path relationships between H2, H8, and H9, all other paths in the hypothesized model are significant. Positive effects are observed among the constructs in all relationship paths, as illustrated in Figure 2.

**Table 9:** Results of path analysis.

Hypothesis	Path	Direct effect		Indirect effect		Total effect		Results
		β	B-C Sig.	β	B-C Sig.	β	B-C Sig.	
H1	CO → FL	0.394	0.003*	/	/	0.394	0.003*	Support
	CO → PU	/	/	0.335	0.003*	0.335	0.003*	
	CO → SA	/	/	0.325	0.003*	0.325	0.003*	
	CO → CI	/	/	0.331	0.003*	0.331	0.003*	
H2	SE → FL	0.046	0.717	/	/	0.046	0.717	Not support
	SE → PU	/	/	0.039	0.712	0.039	0.712	
	SE → SA	/	/	0.038	0.717	0.038	0.717	
	SE → CI	/	/	0.038	0.719	0.038	0.719	
H3	TE → FL	0.597	0.001*	/	/	0.597	0.001*	Support
	TE → PU	/	/	0.508	0.001*	0.508	0.001*	
	TE → SA	/	/	0.493	0.001*	0.493	0.001*	
	TE → CI	/	/	0.501	0.001*	0.501	0.001*	
H4	FL → PU	0.852	0.001*	/	/	0.852	0.001*	Support
H5	FL → SA	0.291	0.022*	0.535	0.001*	0.826	0.001*	Support
H6	FL → CI	0.444	0.003*	0.396	0.003*	0.840	0.001*	Support
H7	PU → SA	0.628	0.001*	/	/	0.628	0.001*	Support
H8	PU → CI	0.294	0.101	0.111	0.205	0.404	0.007*	Not support
H9	SA → CI	0.176	0.238	/	/	0.176	0.238	Not support

* The level of significance is 0.05.

## 5. Discussion

The research findings indicate that control and telepresence have significant positive impact on Flow (H1, H3 are supported). This means that when students explore knowledge in a virtual learning environment independently, they can promote students’ entry into the state of flow by enhancing students’ control ability and improving their telepresence experience. These research results also confirm the views of Rosendahl and Wagner (2023) that the use of SVVR as a teaching medium can enhance the immersive and experiential learning experience. It also validates previous research findings that the use of SVVR devices in art courses can affect students’ learning performance (Wu et al., 2021b). Among them, the β value of telepresence is higher than control (0.597 > 0.394, *p* is 0.001 < 0.003), which means that telepresence is an important factor influencing the state of flow when students use SVVR-assisted materials in art courses, further validating Guo et al. (2016) premise that telepresence is a prerequisite for the flow experience. Therefore, this study believes that emphasizing visual perception (Chang et al., 2022), contextualized learning environments (Gillespie, 2018), and students’ autonomous and creative learning experiences (Wu et al., 2021a), and producing immersion through virtual learning environments (Guo et al., 2016) can help students easily enter the flow state and achieve excellent knowledge acquisition experience when using SVVR teaching materials in art courses.

It is noteworthy that the impact of self-efficacy on flow is not significant (H2 is not supported). This may be due to the limited application of virtual technology in the teaching of art courses for the respondents, with some students using SVVR devices for the first time in the course, requiring a period of adaptation and adjustment during use (Lin et al., 2021). This implies that students may face challenges other than learning new knowledge, leading to feelings of anxiety and lack of confidence, thus affecting their ability to enter a state of flow through self-efficacy.

The flow experience has a positive and significant impact on perceived usefulness, satisfaction, and continuance intention (H4, H5, and H6 are supported). In other words, students who are in a state of flow while using SVVR teaching materials in art courses perceive a higher level of usefulness, satisfaction, and intention to continue using SVVR for learning. This result confirms the positive effect of flow experience on satisfaction in the new media environment (Kim and Ko, 2019; Mulik et al., 2019), highlights the importance of high perceived usefulness in the use of AR/VR technology for virtual learning and teaching (Alalwan et al., 2020; Jang et al., 2021), and shows that students with better flow experiences are more likely to have a higher intention to continue using virtual environment (Li et al., 2022). Compared to traditional teaching methods, SVVR teaching materials provides students with an immersive learning experience that printed materials cannot achieve, which is why students can better enter a state of flow in art courses (Degli et al., 2019; Wu et al., 2021a).

Finally, it was found that SVVR teaching materials, which visually simulates real environments, has a significant impact on continuance intention through perceived usefulness (H7 established). However, there is no direct significant impact of perceived usefulness and satisfaction on continuance intention (H8 and H9 not established). The SVVR teaching materials enables students to perceptually capture more effective information, resulting in a profound understanding of the learning object and a more satisfactory learning effect (Yang et al., 2021). Nevertheless, the efficiency of acquiring knowledge through visual means and the satisfaction of the learning process using SVVR have no influence on continuance intention, potentially due to accumulated fatigue from learning.

## 6. Conclusions and future suggestions

In conclusion, the SVVR teaching materials provide high school art students with a more effective way to enter the state of flow, allowing students to acquire an exceptional immersive virtual learning environment experience, thereby achieving better learning performance. This study further explores the integration of digital art technology into art courses, thus developing new teaching methods that allow students to recognize and perceive art. The conclusions of this article are as follows: (1) as a relatively inexpensive and easy-to-operate virtual reality device, SVVR provides students with a more interactive and more vivid virtual environment learning experience with its high-quality visual information environment; (2) when applying SVVR teaching materials in art courses, more attention should be paid to the control and telepresence in the pre-flow of students, and under the condition of obtaining a stronger flow experience, students will have better perceptual usefulness and satisfaction with the content of art courses, as well as a stronger continuance intention. This study believes that the application of SVVR teaching materials has positively influenced educational methods and teaching concepts, providing possibilities for the promotion of diversified and unique teaching methods. On the other hand, SVVR allows students to have a better self-regulated learning experience in the information-rich virtual world.

Of course, this study also has some limitations. Firstly, it did not cover students from other grades, such as primary and junior high school students. The limited research respondents failed to highlight whether there are differences in the learning effects of SVVR between high school students and students from other grades in art courses. Additionally, there may be differences in the use of SVVR for learning between different genders. Secondly, with the continuous updates and iterations of VR/AR devices, whether SVVR devices can still maintain portability and immersive experience advantages compared to newly updated devices such as Meta Quest Pro or Pico4 Pro, which are highly immersive VR wearable devices, is also a concern. Moreover, with the development of digital technology, the sensory experiences exhibited by MR technology are bound to surpass existing AR/VR technologies. Thirdly, different art courses may show varying learning effects under the support of SVVR teaching materials, such as art field trips and art appreciation courses. The aforementioned research limitations are aspects that need to be explored in future research.

## Data availability statement

The raw data supporting the conclusions of this article will be made available by the authors, without undue reservation.

## Ethics statement

The studies involving human participants were reviewed and approved by Changshu Institute of Technology Ministry of Social Science. Written informed consent to participate in this study was provided by the participants’ legal guardian/next of kin.

## Author contributions

HW and JC: conceptualization and writing—review and editing. DL: methodology, supervision, and project administration. WW: software and data curation. CG and WW: validation. CG: formal analysis, investigation. JC: writing—original draft preparation. JC and WW: visualization. All authors contributed to the article and approved the submitted version.

### Conflict of interest

The authors declare that the research was conducted in the absence of any commercial or financial relationships that could be construed as a potential conflict of interest.

### Publisher’s note

All claims expressed in this article are solely those of the authors and do not necessarily represent those of their affiliated organizations, or those of the publisher, the editors and the reviewers. Any product that may be evaluated in this article, or claim that may be made by its manufacturer, is not guaranteed or endorsed by the publisher.

## Footnotes

^1^ http://www.utovr.com/
